# Correspondence

**Published:** 1861-10

**Authors:** 


### CORRESPONDENCE.

U. S. NAVAL HOSPITAL, N. Y.,
Sept. 33, 1861.

DEAR DOCTOR:—The fortunes of war are proverbially uncertain, and the cruise that I expected so soon to take, when I made my abrupt departure from the Buffalo General Hospital, has been on dry land, consequently I have not put my researches on emesis to the test. I have seen nothing of the war but preparations and results, and am awaiting "active service" with patience, and occasionally desire. The naval victory at Hatteras gave me some clinical experience, but no gun-shot wounds or capital operations. Thirty cases of chronic rheumatism, diarrhœa and dysentery, with other diseases, in small variety, were brought here from the coast by the U. S. Steamers Minnesota and Rhode Island, and they told the whole story of a battle won,

The statistics of this hospital would be of little benefit to the columns of your Journal, inasmuch as we have very few cases of acute or wasting disease, the large majority of patients being men who are returned because unfit for service from broken-down constitutions, or liability to disease in the climate where engaged. At the end of the present month the quarterly report of the patients treated, and their diseases, will be made out, and if it would interest you to contrast it with the reports of civil hospitals, I will give you an abstract.

The hospital, although a very large and fine building, is not well adapted for crowding patients, and two hundred is as many as it will conveniently hold on the single floor, intended for their reception. There have been treated, during the present quarter, more than four hundred patients, the number now remaining being about one hundred and forty. The number of admissions average 25 weekly; the discharges nearly as many.

By changing the topio from patients to doctors, I may, perhaps, better entertain the younger portion of your readers. The Board of Medical Examiners is now in session at this hospital, and daily engaged in the laborious task of testing the qualifications of aspirants to the post of Assistant Surgeons in the U. S. Navy. By a recent act of Congress the corps is to be increased to 80 Surgeons and 120 Assistants and Passed Assistants. This increase will give the Board employment for some time to come. Its previous sessions were to fill vacancies, while the present is for an addition of fifty-one. Twenty-one of these have been selected, consequently there are thirty vacancies yet to fill. The requirements are, that the applicant should be between 21 and 26 years of age, of good physical health and moral character, and in addition he must pass a favorable examination in Anatomy, Chemistry, Medicine, Surgery, &c. On making application to the Secretary of the Navy, stating age and residence, and enclosing certificates of moral character, a permit to appear before the Board will be furnished. Armed with this, and such certificates of character, &c., as a young doctor can obtain, he may come forward for examination. If successful, in a few days he will receive an appointment from the date of acceptance of which his pay commences. The pay of Assistant Surgeons is, at sea, $1250 a year, and ration; on other duty $1050; on leave, or waiting orders, $800.

Names of Assistant Surgeons who have passed the Board of Navy Medical Examiners during the present session:—Adolph A. Hockling, Penn.;

Heber Smith, New York; Stephen I. Clark, New York; Lewis Zinzin, New York; Josiah H. Gunning, New York; Edward Kershner, Maryland; John W. Bragg, Massachusetts; Charles H. Perry, Rhode Island; George R. Brush, New York; Stephen Hugg, New Jersey; T. B. Adams Lewis, New York; Samuel R. Foreman, New Jersey; Watson C. Hull, New York; Daniel Moore Skinner, New Jersey; George T. Shipley, Massachusetts; Charles Ellery Steadman, Massachusetts; William Borrow Mann, New York; Isaac H. Hazelton, New Hampshire; Edward Duggan Payne, Pennsylvania; Benj. H. Ridder, Massachusetts; David F. Rickets, Maryland.

These names are given in the order of time at which they reported, and with no reference to their respective merit. Among them you will recognize the name of William B. Mann, who graduated at the Buffalo Medical College last spring.

Very Respectfully Yours, B.

